# First Report on Fetal Cerebral Polyglucosan Bodies in Mucopolysaccharidosis Type VII

**DOI:** 10.1155/2017/9523427

**Published:** 2017-07-10

**Authors:** Hazim Kadhim, Valérie Segers, Catheline Vilain, Julie Désir, Nicky D'Haene

**Affiliations:** ^1^Neuropathology Unit, Department of Anatomic Pathology and Reference Center for Neuromuscular Pathology, Brugmann University Hospital-Children's Hospital (CHU Brugmann-HUDERF), Université Libre de Bruxelles (ULB), Brussels, Belgium; ^2^Unit of Fetal Pathology, CHU Brugmann-HUDERF, ULB, Brussels, Belgium; ^3^Department of Anatomic Pathology, Erasme Academic Hospital, ULB, Brussels, Belgium; ^4^Center for Medical Genetics, Hôpital Erasme, Université Libre de Bruxelles (ULB), Brussels, Belgium

## Abstract

We report on the detection of discordant inclusions in the brain of a 25-week female fetus with a very rare lysosomal storage disease, namely, Sly disease (mucopolysaccharidosis (MPS) type VII), presenting with nonimmune hydrops fetalis. Besides vacuolated neurons, we found abundant deposition of polyglucosan bodies (PGBs) in the developing brain of this fetus in whom MPS-VII was corroborated by lysosomal beta-glucuronidase-deficiency detected in fetal blood and fetal skin-fibroblasts and by the presence of a heterozygous pathogenic variant in the* GUSB* gene in the mother. Fetal/neonatal metabolic disorders with PGB-deposition are extremely rare (particularly in relation to CNS involvement) and include almost exclusively subtypes of glycogenosis (types IV and VII). The accumulation of PGBs (particularly in the* fetal brain*) has so far* not* been depicted in Sly disease. This is the first report on such “aberrant” association. Besides, the detection of these CNS inclusions at such an early developmental stage is remarkably unique.

## 1. Introduction

Type VII mucopolysaccharidosis (MPS), also known as Sly disease [1], is a* very rare* lysosomal storage disease (LSD) [2], with autosomal recessive (AR) inheritance. Its incidence is estimated between 1 : 300.000 and 1 : 2.000.000 [3] depending on race, ethnicity, and other factors. This rare form of inborn errors of metabolism results from deficiency in the lysosomal enzyme beta-glucuronidase [4]. The clinical picture varies greatly from mild forms (almost unaffected infants) at one end of the phenotypic spectrum, to very severe forms with early neonatal- or even fetal-onset cases. The latter form could present with fetoplacental anasarca, sometimes associated with organomegaly (mainly hepatosplenomegaly) and/or other developmental anomalies (mostly pulmonary and osseous) [5]. MPS type VII has thus been recognized as a cause of nonimmune hydrops fetalis (NIHF) that could be associated with great variability in clinical and biochemical manifestations [4].

It is noteworthy that most newborns with LSDs appear normal at birth because many of the toxic metabolites cross the placenta during pregnancy and are cleared by the mother during gestation; the interval between birth and the onset of clinical symptoms can range from hours to months [4]. The severe neonatal form of MPS-VII is thus among the very few lysosomal storage diseases that might present as early as at birth [1]; it could even be detected in utero (although unfortunately often missed [6]).

Because of the early presentation and the relative severity of many of the estimated 50 different LSDs [4], a correct diagnosis is essential for genetic counselling and subsequent prenatal diagnosis as most of these disorders are AR. In many cases, studies of chorionic villous cells or amniocytes can provide prenatal diagnosis by detecting enzymatic or biochemical changes, namely, in cultured cells. Parallel sequencing of LSDs genes is complementary to the biochemical analysis in most cases and could provide diagnostic confirmation and subtyping.

Clinicopathological changes in LSDs largely result from the accumulation (in various tissues and organs) of defectively catabolized metabolites. In Sly disease, mainly, accumulation of dermatan sulfate and chondroitine classically results in a foamy/vacuolated cell-appearance in affected tissues. First-line diagnosis in* Sly disease* thus depends on assessing these glycosaminoglycans storage-products (previously known as mucopolysaccharides) in biological fluids (urine/amniotic fluid electrophoresis) [7]. Confirmation relies on the detection of the specific enzymatic deficiency, namely, in leukocytes or cultured fibroblasts. Furthermore, DNA diagnostics are becoming increasingly feasible, and the relevant genes can be analyzed. Vacuolization of various cell-types, mainly placental stromal (Hofbauer) cells, has been figured out as the histopathological hallmark in Sly disease, though not pathognomonic to the subclass [1].

To date, classical reviews of metabolic disorders did* not* include MPS type VII (Sly disease) in the group of rare conditions known as “Polyglucosan Body Disorders (PGBD)” [5]. The latter is a heterogeneous group of a few* unrelated* conditions which might occur across the whole age-spectrum [5]. PGBDs have been defined by the presence therein of a common histomorphological denominator, namely, the polyglucosan bodies (PGBs or polyglucosans) in various tissues. The name “PGBs” has actually been used as a generic name [5] to denote bodies that present particular histomorphological and biochemical characteristics. These PGBs therefore characterized those few (but clinically and/or biochemically different) disorders within that PGBD group. And some of these rare disorders display these PGBs inclusively or exclusively in the CNS.

We report the first* fetal* case of Sly disease displaying such PGBs in the developing CNS, and we thus characterize a hitherto nondepicted neuropathological profile in this very rare metabolic lysosomal disorder. We, further, depict the full clinicopathological, morphological, biochemical, enzymatic, and molecular/genetic findings in our case.

## 2. Case Presentation

### 2.1. Case History and Clinical Explorations

A 26-year-old G3P1 pregnant woman presented to our center for second-trimester echography. The healthy couple was nonconsanguineous. Maternal history revealed first-pregnancy medical abortion for conjoint twins, followed by a normal pregnancy and birth of a normal child 3.5 years prior to this latest pregnancy.

*Sonography* revealed generalized fetal hydrops at 23 weeks, with ascites, skin edema (mainly upper body), and mild pericardial effusion but no hydramnios. Maternal blood was Rhesus+ and there was no fetomaternal Rhesus incompatibility. Maternal serology results for TORCH were nonremarkable.

*Amniotic fluid* examination and* cordocentesis* were performed, and search for common infections (possibly implicated in hydrops fetalis (HF)) yielded unremarkable results: CMV direct* microbiological* and* viral culture* results and PCR analyses (CMV, enteroviruses,* Toxoplasma gondii*, parvovirus B19) were negative. Conventional fetal* karyotype* was normal (46; XX), hemoglobin = 9 g/dl, and platelets-count was 87000/mm3.

A* follow-up echography* at 24-weeks showed moderate oligohydramnios with amniotic fluid index at 7 cm.* Biometry* showed liver and abdominal circumference both at *p* > 97.* Umbilical Doppler* results were normal. Importantly, this second sonographic examination revealed a growing HF with increasing ascites (133 cc). A multidisciplinary meeting including the parents and a geneticist was convened to discuss the unfavorable evolution of this gestation with increasing HF/ascites and to further explore the underlying cause. A* second cordocentesis* and* chorionic villi analysis* were contemplated to search for eventual metabolic disorders;* blood tests* were concordant with results obtained from the first examination.

Remarkably,* lysosomal enzymes analyses* (cord blood) showed that beta-glucuronidase activity was undetectable; this was further confirmed by subsequent cultured fibroblasts from fetal skin biopsy. Besides,* amniotic fluid analysis results* showed mild and moderate elevations of dermatan sulfate and chondroitin sulfate, respectively. These results were consistent with MPS type VII (Sly disease) [4, 7].

Given the unfavorable evolution of this pregnancy and the progressing anasarca (HF/ascites),* medical abortion* was induced at 25-week gestation, and a full autopsy was carried out.

All autopsy and neuropathological procedures were conducted in full compliance with ethical rules applied in our institutions.

### 2.2. Autopsy and Auxiliary Explorations

*Autopsic gross examination* of the fetus showed measurements (biometric data) compatible with the given gestational age. Inspection showed female gender and notable anasarca with distended abdomen and skin edema that included the face, limbs, and genitals. There was facial dysmorphism with long philtrum (7 mm) and a distorted external left ear, depressed/flattened nasal bridge, coarse tongue, and short neck. The left fifth toe superimposed the fourth.

*Dissection* revealed ascites (estimated at around 150 ml), pleural and pericardial effusions, pulmonary hypoplasia with lungs/body weight-ratio at 0.005 (normal for age ≥0.015), mild hepatosplenomegaly, left high-ureteral stenosis, and a dilated renal-pelvis. The fixed placenta weighed 580 gm (normal = 190) and presented a peripheral thrombus.

**Figure 1:**
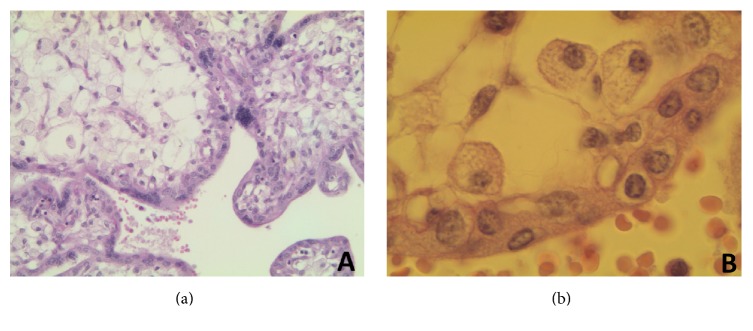
Overview of main histopathological findings. (a) and (b) Placental histopathological results showing a rather loose villous stroma with numerous foamy/vacuolated Hofbauer cells. Periodic Acid-Schiff (PAS) stain; original magnifications: ×200 (a) and ×1000 (b).

*Microscopic examination* showed that the main histopathological finding was that placental villous stroma was rather loose and, remarkably, presented numerous foamy/vacuolated Hofbauer cells (Figures 1(a) and 1(b)). Villi were often voluminous and rather paucivascular. The trophoblast did not show vacuolization. Mild erythroblastosis was noticed. Examination did not show abnormal interstitial foamy cells in hepatic-splenic tissues or the other dissected thoracoabdominal organs. Subtle vacuolization of few cardiac myocytes was noted.

*CNS examination*: The postfixed whole brain weighed 114 g, and the infratentorial segment (brainstem/cerebellum) weighed 5.9 g. Inspection showed recent pericerebellar hemorrhage. There were no gross anomalies and the different cerebral structures presented a developmental aspect compatible with gestational age. Examination of serial coronal sections of the cerebral hemispheres and of horizontal sections of the infratentorial structures showed residual ventricular hemorrhage, namely, in the lateral ventricle.

*Microscopic examination* of the brain showed a normally developing cerebral wall (pallium), with a distinct cortical plate, intermediate zone (incipient white matter), and a notable periventricular germinal zone, concordant with gestational age. There were a generalized vascular congestion and foci of microspongiosis in the cerebral white matter (WM). The cerebellum also was developmentally normal for age with an external granular layer, a thin molecular zone, Purkinje cells-related lamina dissecans, and an emerging internal granular layer [8].

**Figure 2:**
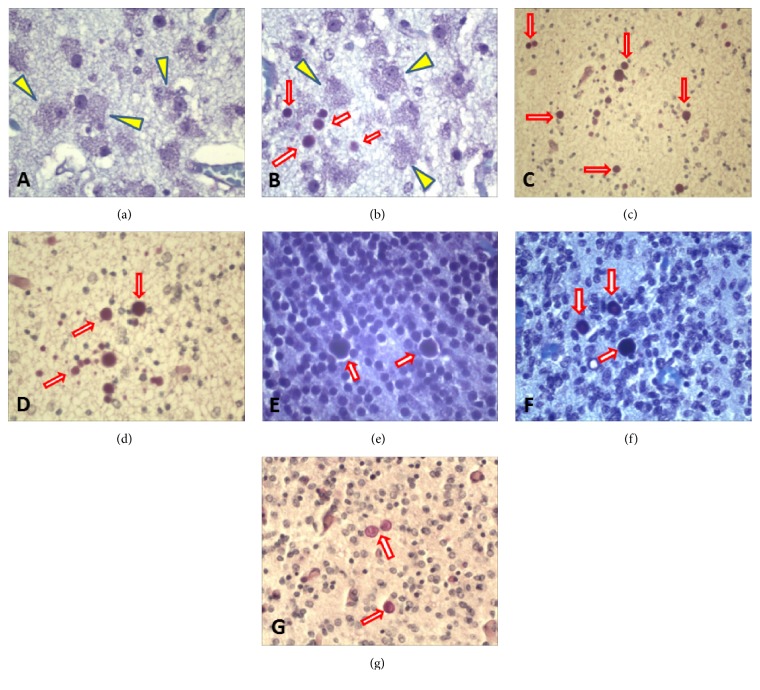
Major neuropathological findings in our case of Sly disease. ((a) and (b)) Histopathological sections from the brain showing numerous neuronal cells with remarkably foamy/vacuolated perikaryonal cytoplasm (arrow-heads; yellow) in the striatum of this 25-year-old fetus. Note also the presence of PGBs (arrows in (b)) in the neuropil. (b), (c), (d), (e), (f), and (g) illustrate the histomorphological appearance and staining characteristics of the many PGBs (some of which indicated by arrows) detected in various brain structures of this fetus, namely, the incipient cerebral white matter ((c) and (d)), the developing cortical plate of the neocortex (e), the telencephalic periventricular germinal zone (f), the striatum (b), and the developing cerebellum (g). This figure, besides, shows that at all levels of the brain, all PGBs appear to be deposited in the neuropil, and none actually look clearly intracellular with any of the various histopathological techniques we employed ((c), (d), (e), (f), and (g)). ((a) through (g)) Light microscopic photographs, Cresyl-violet (Nissl) staining ((a), (b), (e), and (f)), Hematoxylin-Eosin staining (g), and Periodic Acid-Schiff/D-amylase (PAS-D) staining ((c) and (d)). Original magnification: ×400 ((a), (b), (d) and (f)), ×250 (c), ×500 (e), and ×320 (g).

Remarkably, most neuronal cells in the cerebral* deep* gray matter, notably the striatum, presented a* foamy* perikaryonal cytoplasm (Figures 2(a) and 2(b)). The most striking histomorphologic observation, however, was the presence of numerous opaque/hyaline or concentrically laminated deposits in the brain that were ovoid or globular, although some elongated or semiring forms could also be found. Outline occasionally looked irregular, and the center sometimes showed higher density. These bodies had variable sizes (mostly between 5 and 30 micrometers) and presented varying degrees of basophilia on Hematoxylin-Eosin (HE) staining (blue/gray). They were additionally characterized by a strong Periodic Acid-Schiff- (PAS-) positive reaction (dark red) that was resistant to diastase (PAS-D) treatment. These structures thus presented staining and histomorphological characteristics typical of PGBs (Figures 2(b), 2(c), 2(d), 2(e), 2(f), and 2(g)). Topographically, PGBs were detected in the different layers of the developing cerebral wall: they were more abundant in the cerebral WM (Figures 2(c) and 2(d)). Many PGBs were, however, also seen in the cortical plate of the developing neocortex (Figure 2(e)), in the deep cerebral gray matter (Figure 2(b)), and in the telencephalic periventricular germinal zone (Figure 2(f)), though in lesser numbers than in the WM. A few were also found in the allocortex/hippocampal formation and in the cerebellum (Figure 2(g)). The outermost layer of the cerebral wall, namely, the subpial molecular zone, was the only stratum where these deposits could not be detected. It is noteworthy that at all levels of the brain examined in this unique case, practically all these PGBs, appeared to be deposited in the neuropil of the various sites described, and none actually looked clearly intracellular as portrayed with the various histopathological techniques we employed (Figures 2(c), 2(d), 2(e), 2(f), and 2(g)). This neuropil-localization was actually in line with a previous report published on PGBs in a related CNS disorder [9]. Eventual electron microscopic explorations might further contribute to additional positional (and structural) characterization.

### 2.3. Supplementary Explorations

*Enzymatic analysis* of beta-glucuronidase activity in the parents showed results compatible with (though not diagnostic of) a carrier state: 15 nmol/ml/h in the father and 21 nmol/ml/h in the mother (normal range = 11–154 nmol/ml/h, mean = 54 nmol/ml/h, and control value = 60 nmol/ml/h).

*Genetic studies*: targeted-PCR and sequencing of both DNA strands of the entire coding regions and exon-intron splice junctions were carried out in the parents (Centogene, Rostock, Germany). Results showed a heterozygous c.1120C>T variant, leading to a R374C substitution within exon 7 of the* GUSB* gene in the mother. This variant has already been identified as pathogenic in MPS-VII [10], and the mother's results were thus indicative of a carrier status for MPS-VII. Genetic analysis in the father did not show nucleotide substitution, but additional techniques to rule out possible deletion of one or more exons were not performed. Genetic studies in the* fetus* focused on conventional karyotype analysis (that was unremarkable) with no additional molecular explorations.

## 3. Discussion

Fetal and neonatally detected metabolic disorders of genetic basis with polyglucosan bodies (PGBs) are extremely rare and are nonreported in relation to CNS involvement. The rare conditions that present PGBs (at this age-group) include mainly glycogenosis IV (and probably type VII [5]), in which cases these PGBs are almost exclusively restricted to striated muscles [11]. The accumulation of PGBs, particularly in the* fetal brain,* has* not* been so far depicted in the very rare cases of Sly disease. This observational report is the first on such a finding. Moreover, the detection of these CNS inclusions at such an early developmental stage is remarkably unique.

Notably, this fetal brain presented, in addition to the PGBs, numerous vacuolated neuronal cell bodies. The presence of such vacuolated neurons has* not* been, either, clearly depicted in the CNS at such an early developmental stage in Sly disease. In this metabolic disorder, characteristic foamy cells, namely, macrophages/histiocytes, prevail in the placenta. However, related or possibly different cells with foamy or vacuolated cytoplasm have occasionally been also portrayed in few other (extracerebral) sites. It is perhaps noteworthy here that the full extent of cells' affection and of organs' involvement in this disorder is not yet fully revealed. This is particularly so in cases of* early fetal* stages (and more so in the* brain*) and is (perhaps not unexpectedly) mainly due to the extreme rarity of the condition. Reportedly (see Molyneux et al. [12] for review), the degree and distribution of cellular vacuolization within organs appears to be variable. Furthermore the precise nature of these vacuolated cells have mostly not been clearly identified and could seemingly involve various cell-types (as has been pointed out also in “Introduction”). Comprehensive immune-cytological explorations on abnormal cells in various affected sites/organs could help provide further characterization.

The unexpected detection in our case of “aberrantly discordant” CNS parenchymal inclusions (whose staining and histomorphological characteristics were typical of PGBs) was particularly unique and actually puzzling. This hitherto nonreported association is thus exceptional, and the unexpected detection of cerebral PGBs raises the question whether these inclusions constitute a previously unknown feature of MPS type VII. The full spectrum of neuropathological findings in Sly disease might thus need to be further ascertained.

On the other hand, the concurrence of a second genetic disease remains possible (though very rare) in infants with known inherited metabolic diseases. In our case, however, meticulous reexamination of all other body-organs did not reveal signs of an associated PGB-disease as there were no such inclusions elsewhere in the body.

Our observation on CNS PGBs thus highlights the importance of additional meticulous explorations by expert fetal neuropathologists in all cases of Sly disease to further assess the extent and prevalence of PGs in this condition: If such association is substantiated in more cases, Sly disease can then be added to the small group of disorders included under the umbrella term “polyglucosan disorders” [5]. The latter is a group of diverse conditions (with PGB inclusions) in which the individual disorders might present at different times of life.

PGB accumulation is best known in the exceptionally rare* infantile* metabolic disorders of glycogenosis (almost exclusively type IV [1] and possibly type VII [5], in which CNS involvement, though, still remains to be clearly shown). Similar inclusions were also found in other (albeit very limited number of) inherited metabolic disorders that affect age-groups* beyond infancy* (i.e., childhood and adolescence), namely, Lafora disease [5]. More rarely, PGBs have been also reported in pathological disorders with CNS involvement in* adult* patients [9, 13, 14], namely, in ALS-like athetosis and Adult Polyglucosan Body Disease (APGBD) [5, 9, 13, 14]. The latter is a heterogeneous group (both genetically and clinically) of CNS disorders with at least more than one biochemical basis [13, 14] and includes a myopathic subtype, so-called “polyglucosan body myopathy” [11], and another subtype called “polyglucosan axonal neuropathy.”

Our present report might thus* expand the spectrum* of this group so-called “polyglucosan disorders” [5] that would thus span all age-groups and provides further evidence for the heterogeneous origin of this small group of disorders with PGBs.

This report also exemplifies the impact of expert prenatal/perinatal diagnostic approaches [2, 7, 12, 15] (including detailed neuropathological and genetic/molecular explorations) on proper diagnostic outcome particularly in identifying eventual rare inherited metabolic disorders. It crucially shows how* adequate awareness* of “unusual association” of clinicopathological features could ensure no missing of the wider spectrum in a given nosological context (particularly in rare or newly detected conditions) and may help to reduce misdiagnosis.

Finally, it is noteworthy that, given the extreme rarity of Sly disease, biomedical researchers contemplating advancements in pathophysiological mechanisms underlying this genetic disorder managed to develop animal-models, mainly murine/mouse types of the condition. Histomorphological studies in these animal-models suggested, like in the human [12], the presence of a wide spectrum of cytohistopathological variations regarding the degree, distribution, and type of cells' and organs' involvement (see Kumar et al. [16] for review and references). Comparative studies from these animal and human observations thus suggest that “observed” inter- and intraspecies differences or discrepancies farther widen when both early-onset (fetal, neonatal, juvenile, etc.) and more mature cases were involved. As a corollary and because of the observed diversity and the notable differences (in the degree of cells' and organs' involvement (in both human or animal-models)) which could even become more pronounced if sampling covers the whole life-spectrum (chronicity/endurance of disease), comparative histomorphological studies (including neuropathological) would therefore* expectedly* portray a rather heterogeneous set of observations that could look mostly unrelated (in structure, morphology, topographic localization, and even etiopathogenesis (because of possible activation of secondary physiopathological pathways like inflammation, ischemia, etc.)).

Lumping of the observed plethora of differences “under common denominators” or attempts for further categorizations would be of limited feasibility at the current stage of knowledge because of the yet incomplete knowledge of the full spectrum of the cellular/structural changes and also because of the extreme rarity of this condition in humans (particularly in relation to the extreme paucity of detailed neuropathological data in* early* prenatal cases!).

These, mainly mice animal-models, also served for contemplating eventual therapeutic approaches, using mainly enzyme replacement- and gene-therapies. Some promising (though mostly limited) results were sometimes reported and could provide reasonable hope for forthcoming clinical “cure.” Despite these preclinical outcomes, translation of those therapies into the clinic, however, lagged behind other LSDs, largely because of the very rare incidence of MPS-VII. This highlights again the crucial importance of detailed case-reporting on this extremely rare metabolic disorder, particularly regarding the full spectrum of CNS involvement (and more so in the early fetal cases).

## Abbreviations

CMV: Cytomegalovirus
CNS: Central nervous system
CP: Cortical plate, cerebral
Hb: Hemoglobin
HE: Hematoxylin-Eosin (staining)
HF: Hydrops fetalis
LSD: Lysosomal storage diseases
MPS: Mucopolysaccharidosis
NIHF: Nonimmune hydrops fetalis
PAS: Periodic Acid-Schiff (staining)
PAS-D: Periodic Acid-Schiff -diastase/amylase (staining)
PCR: Polymerase chain reaction
PG: Polyglucosans
PGB: Polyglucosan bodies
PGBD: Polyglucosan Body Disease(s)/Polyglucosan Body Disorder(s)
APGBD: Adult Polyglucosan Body Disease
WM: White matter, cerebral.

## Additional Points

*Highlights*. (i) Sly disease is a very rare recessive metabolic disorder, classically with vacuolated cells. (ii) Reports on* prenatal CNS involvement* are extremely rare. (iii) Presence of “polyglucosan bodies” in fetal CNS has never been reported. (iv) This is the* first report* on fetal cerebral polyglucosans in Sly disease. (v) Awareness of unusual clinicopathological associations helps recognize the wider spectrum in a given nosological context (particularly in rare or newly detected conditions) and helps lessen misdiagnosis.
